# Supplementary material for: WordCommentsAnalyzer: A windows software tool for qualitative research
Source: F1000Res. 2018 Sep 4;7:536. Originally published 2018 May 3. [Version 2] doi: 10.12688/f1000research.14819.2 (PMC6137411; doi:10.12688/f1000research.14819.2)
Supplement: Supplementary file 3 [file f1000research-7-17614-s0002.tgz › b2a79496-4ada-47c4-9682-a99ce867836d.zip!/Eight Iranian Health Journals/Archives of Rehabilitation_37.docx]

© Archives of Rehabilitation. This work is licensed under CC BY-NC 4.0 (https://creativecommons.org/licenses/by-nc/4.0/).

Volume & Issue: دوره 11 - شماره 3

Title: مقایسه سواد خواندن در دانش‌آموزان آسیب‌دیده شنوایی در سه مقطع تحصیلی

http://rehabilitationj.uswr.ac.ir/article-1-637-fa.html

هدف: پژوهش حاضر با هدف بررسی پیشرفت سواد خواندن در دانش‌آموزان آسیب‌دیده شنوایی و مقایسه سواد خواندن آنان در سه مقطع تحصیلی انجام پذیرفت. روش بررسی: در این تحقیق تحلیلی و کاربردی که از نوع مقطعی-مقایسه‌ای می‌باشد، از بین جامعه آماری شامل دانش‌آموزان پایه چهارم ابتدایی، سوم راهنمایی و سوم دبیرستان مدارس استثنایی شهرستانهای کرج، شهریار، شهرقدس، هشتگرد و رباط‌کریم، ۱۱۹ دانش‌آموز آسیب‌دیده شنوایی به‌روش در دسترس انتخاب و توانایی خواندن و درک متون اطلاعاتی و ادبی آنها با استفاده از ابزار پژوهش یعنی دفترچه‌های مطالعه بین‌المللی پیشرفت سواد خواندن (پرلز) مورد سنجش قرار گرفت. داده‌های حاصل با استفاده از آزمون تی مستقل برای مقایسه گروه‌ها تحلیل شد. یافته‌ها: بین پایه‌های تحصیلی چهارم ابتدایی و سوم راهنمایی با آسیب شنوایی شدید و متوسط، سواد خواندن و درک متون اطلاعاتی و ادبی اختلاف معناداری نداشت (0/05 نتیجه‌گیری: پیشرفت سواد خواندن در دانش‌آموزان آسیب‌دیده شنوایی روند بسیار کندی دارد که فراتر از مقطع تحصیلی و میزان کاهش شنوایی می‌باشد. لزوم توجه به ارتقاء سواد خواندن و هدایت به‌سمت لایه‌های عمیق یادگیری و درک مطلب در مقاطع مختلف تحصیلی در سیستم آموزش استثنایی شایان توجه است.

Title: بررسی پایایی و روایی پرسشنامه سرشت و منش

http://rehabilitationj.uswr.ac.ir/article-1-638-fa.html

هدف: پرسشنامه سرشت و منش برای ارزیابی ابعاد شخصیت که مطابق مدل زیستی- روانی- اجتماعی کلونینگر دارای دو بعد سرشت (شامل نوجویی، آسیب‌پرهیزی، پاداش- وابستگی و پشتکار) و منش (شامل خود- راهبری، همکاری و خود-فراروی) می‌باشد تهیه شده است. هدف این پژوهش بررسی پایایی و روایی این پرسشنامه بود. روش بررسی: در این پژوهش که از نوع اعتبارسنجی و آزمون‌سازی است، پس از برگردان پرسشنامه به فارسی، فرم نهایی آن بر روی 220 دانشجو که به روش نمونه‌گیری تصادفی ساده انتخاب شده بودند، اجرا گردید. بررسی پایایی پرسشنامه به‌کمک روش آلفای کرونباخ و بازآزمایی، تأیید روایی آن با بهره‌گیری از تحلیل عاملی چرخش پروماکس، همبستگی درونی مقیاس‌ها و همبستگی سن با مقیاس‌ها با روش پیرسون و مقایسه نمره‌های پرسشنامه بین دو جنس و نیز مقایسه بین‌فرهنگی با آزمون تی‌مستقل انجام شد. یافته‌ها: دامنه ضریب آلفا از 0/44 برای مقیاس پشتکار تا 0/81 برای مقیاس خودفراروی به‌دست آمد. میانگین ضریب‌های به‌دست‌آمده 68/0 و ضریب آلفای کل آزمون 0/74 بود. دامنه ضریب‌های همبستگی پیرسون به روش بازآزمایی بر روی 31 دانشجو به فاصله دو ماه از 0/53 برای مقیاس‌های نوجویی و پشتکار تا 0/82 برای مقیاس آسیب‌پرهیزی و از 0/24 برای خرده‌مقیاس بی‌نظمی در برابر نظم و ترتیب تا 0/86 برای خرده‌مقیاس ترس از عدم موفقیت در برابر اعتماد به نفس در نوسان بود. در تحلیل عاملی 6 عامل به‌دست‌آمد. همبستگی بین مقیاس‌های خود- راهبری و آسیب‌پرهیزی 0/57 و خود- راهبری و همکاری 0/46 به‌دست آمد. نتیجه‌گیری: پرسشنامه سرشت و منش از ویژگیهای روانسنجی رضایت‌بخش و پایایی و روایی قابل‌قبولی برای استفاده در جمعیت دانشجویان دانشگاه برخوردار است.

Title: علل ژنتیکی عقب‌ماندگی ذهنی در استان گلستان

http://rehabilitationj.uswr.ac.ir/article-1-639-fa.html

هدف: سهم عوامل ژنتیکی در بروز عقب‌ماندگی ذهنی حدود 70% است. با توجه به این که شناخت علل ژنتیکی بروز این عارضه، موجب برنامه‌ریزی بهتر و انجام مشاوره دقیق‌تر در جهت پیشگیری و کنترل آن می‌باشد، لذا هدف از این مطالعه بررسی علل ژنتیکی عقب‌ماندگی ذهنی در استان گلستان می‌باشد. روش بررسی: در این پژوهش توصیفی که از نوع مطالعات مقطعی- کاربردی است، با همکاری بهزیستی استان گلستان، خانواده‌های دارای فرزند عقب‌مانده ذهنی شناسایی و 50 خانواده از نقاط مختلف استان که دارای تعداد 2 و یا بیشتر فرزند عقب‌مانده ذهنی بودند با روش نمونه‌گیری ساده انتخاب شدند. عقب‌ماندگی ذهنی افراد مبتلا قبلاً با انجام مشاوره ژنتیک و معاینه بالینی توسط پزشک تأیید شده بود. نمونه‌گیری از خون کلیه افراد مبتلا و سالم در خانواده انجام گرفت. ضمناً در مورد بیماران، بررسی بدشکلی (دیسمورفیسم) و سنجش میکروسفالی (کاهش در اندازه دور سر) انجام شد. سپس افراد مبتلا تحت بررسی کروموزومی (سیتوژنتیک)، شکنندگی کروموزوم ایکس (نشانگان ایکس شکننده)، تست متابولیک و آنالیز پیوستگی برای هفت جایگاه ژنی شناخته شده عقب‌ماندگی ذهنی اتوزومی مغلوب همراه با میکروسفالی (ام.سی.پی.اچ.) قرار گرفتند. یافته‌ها: ناهنجاری کروموزومی در هیچ یک از خانواده‌های مورد مطالعه دیده نشد. از میان 50 خانواده مورد بررسی، یک خانواده مبتلا به نشانگان ایکس شکننده و10 خانواده مبتلا به عقب‌ماندگی ذهنی همراه با میکروسفالی بودند. از میان 10 خانواده میکروسفال، 5 خانواده به جایگاه‌های ژنی ام.‌سی.‌پی.‌اچ. پیوستگی نشان دادند. نتیجه‌گیری: عقب‌ماندگی ذهنی اتوزومی مغلوب همراه با میکروسفالی با سهمی حدود 20 درصد، میزان بالایی از عقب‌ماندگی ذهنی ژنتیکی را در استان گلستان به خود اختصاص داده است.

Title: تأثیر خودآموزش‌دهی کلامی بر بهبود نشانه‌های بی‌توجهی در دانش‌آموزان مقطع ابتدایی مبتلا به اختلال کمبود توجه

http://rehabilitationj.uswr.ac.ir/article-1-640-fa.html

هدف: اختلال نارسایی توجه- بیش‌فعالی یکی از متداول‌ترین شرایط مزمن روانی در خلال کودکی است که با نشانه‌های بی‌توجهی، بیش‌فعالی و برانگیختگی مشخص می‌شود. پژوهش حاضر با هدف بررسی تأثیر خودآموزش‌دهی کلامی بر بهبود نشانه‌های بی‌توجهی در دانش‌آموزان مقطع ابتدایی مبتلا به اختلال نارسایی توجه- بیش‌فعالی، نوع بی‌توجهی غالب انجام شد. روش بررسی: پژوهش حاضر از نوع تجربی (آزمایشی) با طرح پیش‌آزمون و پس‌آزمون و گروه کنترل بود. از جامعه آماری متشکل از دانش‌آموزان پایه‌های دوم و سوم مقطع ابتدایی شهر تبریز، با استفاده از روش نمونه‌گیری خوشه‌ای، 30 دانش‌آموز پسر مبتلا به اختلال نارسایی توجه- بیش‌فعالی، نوع بی‌توجهی غالب بر مبنای نسخه چهارم پرسشنامه نشانه مرضی کودکان (سی اس آی-۴) و مصاحبه روانشناس بالینی (مبتنی بر ملاکهای دی‌اِس‌اِم-۴) انتخاب و بر اساس آزمون هوش ریون رنگی همتا شده و به‌طور تصادفی در دو گروه آزمایش و کنترل قرار گرفتند. گروه آزمایش در 8 جلسه خودآموزش‌دهی کلامی را دریافت نمودند. به‌منظور بررسی ارتباط نشانه‌های بی‌توجهی و خودآموزش‌دهی کلامی و کنترل اثر پیش‌آزمون، از تحلیل کوواریانس استفاده شد. یافته‌ها: نتایج تحلیل کوواریانس نشان داد که بین کاربندی خودآموزش‌دهی کلامی و کاهش نشانه‌های بی‌توجهی رابطه معنادار (P<0.001) وجود دارد. نتیجه‌گیری: مداخله خودآموزش‌دهی کلامی باعث کاهش نشانه‌های بی‌توجهی دانش‌آموزان مبتلا به اختلال بی‌توجهی غالب شد.

Title: مقایسه کودک‌آزاری بین کودکان عادی وکودکان دارای اختلال یادگیری در مقطع ابتدایی

http://rehabilitationj.uswr.ac.ir/article-1-645-fa.html

هدف: تحقیق حاضر به‌منظور مقایسه کودک‌آزاری در کودکان عادی و کودکان دارای اختلال یادگیری 7 تا ۱۲ ساله شهر تهران انجام گرفت. روش بررسی: این مطالعه تحلیلی و مقطعی، از نوع علی – مقایسه‌ای می‌باشد. به روش نمونه‌گیری خوشه‌ای چند مرحله‌ای ۱۲۰ دانش‌آموز عادی از مناطق 3، 7 و 15 آموزش و پرورش شهر تهران و ۱۲۰ دانش‌آموز دارای اختلال یادگیری از سه مرکز 1، 2 و3 انتخاب شده و با استفاده از پرسشنامه معتبر کودک‌آزاری تحت ارزیابی قرار گرفتند. داده‌های حاصل با استفاده از ضریب همبستگی پیرسون و آزمون رتبه‌ای فریدمن و آزمونهای تی‌زوجی و تی مستقل تجزیه و تحلیل شد. یافته‌ها: تفاوت معناداری در میانگین نمرات آزار عاطفی (۰/۰۰۱>P) و نمره کل (۰/۰۰۲=P) کودک‌آزاری به‌دست آمده از پاسخ‌های کودکان، در بین دو گروه کودکان عادی و کودکان دارای اختلال یادگیری مشاهده شد. مطابق نظرات والدین نیز تفاوت معناداری بین میانگین نمرات آزار عاطفی (۰/۰۰۱>P)، آزار جسمی (۰/۰۱۱=P) و نمره کل کودک‌آزاری (۰/۰۰۱>P) دو گروه کودکان عادی و کودکان دارای اختلال یادگیری به‌دست آمد. همچنین تفاوت معناداری بین نظرات کودکان با والدینشان در مؤلفه آزار جسمی (۰/۰۰۲=P)، آزار جنسی (۰/۰۰۱>P) و غفلت (۰/۰۰۱>P) وجود داشت. نتیجه‌گیری: با توجه به نتایج حاصل به‌نظر می‌رسد که بین میزان کودک‌آزاری برحسب نوع در کودکان عادی و کودکان دارای اختلال یادگیری تفاوت وجود دارد و برخلاف گذشته، بیشتر از نوع آزار عاطفی است. بنابراین ضروری است متخصصان بهداشت روان، برنامه‌هایی به‌منظور آموزش والدین در آینده تدارک ببینند.

Title: مقایسه دانش‌آموزان آسیب‌دیده شنوایی شدید تلفیقی و دانش‌آموزان شنوای مقطع راهنمایی در درک ترکیبات استعاری

http://rehabilitationj.uswr.ac.ir/article-1-646-fa.html

هدف: محققین معتقدند افراد آسیب‌دیده شنوایی، آسیب ویژه‌ای در درک استعاره‌ها دارند. مطالعه حاضر با هدف تعیین منشاء آسیبهای درک استعاره در این کودکان و این که آیا ناشی از محیط آموزشی ایشان می‌باشد، به مقایسه درک ترکیبات استعاری دانش‌آموزان آسیب‌دیده شنوایی تلفیقی شدید مقطع راهنمایی با همکلاسی‌های شنوای آنها می‌پردازد. روش بررسی: در این مطالعه مقطعی – مقایسه‌ای، 50 دانش‌آموز شنوا و25 دانش‌آموز آسیب‌دیده شنوایی تلفیقی به روش تصادفی ساده انتخاب شده و از طریق دو تکلیف محقق‌ساخته تحت ارزیابی قرار گرفتند. ابتدا تکلیف درک واژگان غیراستعاری اجرا می‌شد. درصورت پاسخ صحیح به بیش از 75 درصد کلمات و دارا بودن بقیه معیارهای انتخاب نمونه، تکلیف درک ترکیبات استعاری اجرا می‌شد. سپس داده‌ها با استفاده از آزمون‌های آماری تی مستقل و آنالیز واریانس مورد تجزیه و تحلیل و مقایسه بین دو گروه قرار گرفت. یافته‌ها: نمره درک ترکیبات استعاری دانش‌آموزان آسیب‌دیده شنوایی تلفیقی نسبت به دانش‌آموزان شنوا به‌طور معناداری کمتر بود (001/0>P). تعداد پاسخهای غلط لفظی دانش‌آموزان آسیب‌دیده شنوایی تلفیقی بیشتر از پاسخهای غلط معنایی بود. میانگین پاسخهای صحیح بین سه پایه تحصیلی در آزمودنی‌های شنوا (0/401=P) و همچنین دانش‌آموزان آسیب‌دیده شنوایی تلفیقی (0/120=P) اختلاف معناداری نداشت. نتیجه‌گیری: پاسخ‌های غلط دانش‌آموزان آسیب‌دیده شنوایی به آزمون، نشانی از درک ناکامل ایشان از ترکیبات استعاری است و تفاوت فاحش دانش‌آموزان شنوا و آسیب‌دیده شنوایی تلفیقی در درک ترکیبات استعاری، نشان می‌دهد که محیط آموزشی مدارس تلفیقی به‌تنهایی نمی‌تواند در درک این ترکیبات مؤثر باشد.

Title: مقایسه تغییرپذیری زمان‌بندی تنظیمات وضعیتی پیش‌بینانه در بیماران مبتلا به کمردرد غیراختصاصی تکرارشونده و افراد سالم

http://rehabilitationj.uswr.ac.ir/article-1-647-fa.html

هدف: این مطالعه با هدف بررسی تغییرپذیری استراتژی‌های کنترل وضعیت در بیماران مبتلا به کمردرد غیراختصاصی تکرارشونده، به‌عنوان شاخصی در ارزیابی قابلیت تطابق سیستم کنترل و ثبات وضعیت با نیازهای محیطی و مقایسه آن با افراد سالم و همچنین توجه به نقش عوامل روانشناختی همراه با درد به‌عنوان یک عامل تأثیرگذار در اختلال وضعیت انچام شد. روش بررسی: این تحقیق تحلیلی، مقطعی و موردی- شاهدی، بر روی ۲۱ بیمار مبتلا به کمردرد غیراختصاصی تکرارشونده که به‌صورت ساده و در‌دسترس و ۲۱ فرد سالم که به روش جورکردن، از طریق همتاسازی با بیماران انتخاب شده بودند، انجام شد. فعالیت الکترومیوگرافی عضلات سه‌گوش، مایل خارجی و مایل داخلی/ عرضی شکم و راست‌کننده ستون‌مهره‌ها در طی حرکت پرتابی فلکشن بازو با حداکثر شتاب، به تعداد ۷۵ مرتبه در هر فرد ثبت گردید. جهت بررسی باورهای اجتنابی افراد، از پرسشنامه استاندارد باورهای اجتنابی ناشی از ترس و جهت ارزیابی ناتوانی ناشی از کمردرد از پرسشنامه رولند- موریس استفاده گردید. داده‌های حاصل با استفاده از تحلیل واریانس چندگانه و ضریب همبستگی پیرسون مورد تجزیه و تحلیل آماری قرار گرفت. یافته‌ها: بررسی نتایج نشان داد که افراد مبتلا به کمردرد مزمن به‌طور معناداری، تغییرپذیری کمتری در زمان‌بندی تنظیمات وضعیتی پیش‌بینانه عضله عرضی شکم / مایل داخلی نسبت به افراد گروه کنترل نشان می‌دهند(0/047=P)، درحالی‌که کاهش تغییرپذیری محسوس و معناداری در عضلات مایل خارجی(0/45=P) و راست‌کننده ستون‌مهره‌ها(0/6=P) در آنها مشاهده نگردید. بین تغییرپذیری زمان‌بندی تنظیمات وضعیتی پیش‌بینانه و نمره پرسشنامه ترس از درد(0/006=P) ارتباط معناداری وجود داشت، هرچند ارتباط معناداری بین این متغیر و ناتوانی(0/90=P) در بیماران مشاهده نگردید. نتیجه‌گیری: کاهش تغییرپذیری در سیستم کنترل وضعیت بیماران مبتلا به کمردرد غیراختصاصی تکرارشونده وجود دارد که می‌تواند با کاهش قدرت تطابق با نیازهای محیطی موجود، به تداوم کمردرد و مزمن شدن آن بیانجامد. بهتر است بازگرداندن تغییرپذیری به سیستم کنترل وضعیت نیز در توانبخشی این گروه بیماران مدنظر قرار گیرد.

Title: مقایسه ارتباط بین اجزاء حافظه فعال و درک جمله در بیماران مبتلا به آلزایمر و سالمندان سالم

http://rehabilitationj.uswr.ac.ir/article-1-648-fa.html

هدف: دشواری در کسب اطلاعات جدید معمولا اولین نشانه خاموش‌‌‌ در بیماریهای پیشرونده مغزی نظیر آلزایمر است. در این بیماری فرایندهای شناختی دیگر نظیر درک جمله نیز دچار اشکال می باشند. هدف پژوهش حاضر بررسی ارتباط بین حافظه فعال و درک جمله در بیماران مبتلا به آلزایمر و مقایسه آن با سالمندان سالم می باشد. روش بررسی: نوع مطالعه مقطعی، تحلیلی- مقایسه ای است. با مراجعه به انجمن آلزایمر ایران 10 بیمار مبتلا به آلزایمر از بین افراد دارای 60 سال تمام به بالا و 10 سالمند سالم به صورت تصادفی ساده انتخاب شده و با انجام مصاحبه و تکمیل پرسشنامه با بیماران مبتلا و خانواده های آنها، آزمون مختصر وضعیت شناختی، آزمونهای حافظه فعال و درک جمله در مورد آنها اجرا گردید. داده های حاصل با استفاده از ضریب همبستگی پیرسون و آزمون تی مستقل تجزیه و تحلیل شد. یافته‌ها: نتایج نشان داد که دو گروه در آزمون های مدار واجی مربوط به حافظه فعال تفاوت معنادار نشان ندادند (P=0/480). ، اما در آزمونهای اجراکننده مرکزی حافظه فعال تفاوت برجسته نشان دادند (P=0/006). در متغیر درک جملات ساده P=0/001 و پیچیده (P=0/004) تفاوت دو گروه معنادار بوده است. بین متغیر مدار واجی با درک جملات ساده (r=0/524, P=0/120) و پیچیده (r=0/790 P=0/007)، اجراکننده مرکزی با درک جملات ساده (r=0/847, P=0/001) و پیچیده (r=0/667, P=0/035) در گروه بیماران مبتلا به آلزایمر، همبستگی مستقیم و ناقص وجود دارد. نتیجه‌گیری: یافته های فوق نشان می دهد که بیماران مبتلا به آلزایمر در مقایسه با سالمندان سالم، در ظرفیت حافظه فعال کاهش نشان می دهند. همچنین اختلال درک جمله در این بیماران مربوط به منابع کاهش یافته حافظه فعال می باشد که با اجراکننده مرکزی همبستگی بالایی دارد.

Title: تأثیر استفاده از گردنبند طبی بر تعادل ایستادن در حالتهای مختلف سر و گردن

http://rehabilitationj.uswr.ac.ir/article-1-661-fa.html

هدف: علیرغم استفاده گسترده از گردنبند طبی، هنوز اطلاعات کاملی از تأثیر احتمالی آن بر روی ثبات وضعیتی وجود ندارد. هدف این مطالعه بررسی تأثیر استفاده از گردنبند طبی در وضعیت‌های خنثی (نوترال) و «خم‌کردن به‌عقب» (اکستانسیون) سر و گردن بر روی تعادل ایستادن است. روش بررسی: در این مطالعه مداخله‌ای شبه تجربی، 36 دانشجوی سالم به‌روش ساده از نمونه‌های در دسترس انتخاب و میانگین جابجایی و سرعت نوسان مرکز فشار در جهت جلو- عقب، جانبی و کل، با استفاده از صفحه نیرو در 4 وضعیت مختلف ایستادن شامل وضعیت خنثی و20 درجه خم سر و گردن به عقب، با استفاده از گردنبند طبی و بدون آن اندازه‌گیری گردید. داده‌های حاصل با استفاده از آزمون‌ آماری تی‌زوجی مورد تجزیه و تحلیل قرار گرفت. یافته‌ها: سرعت نوسان جانبی در وضعیت 20 درجه خم‌شدن سر و گردن به عقب با استفاده از گردنبند طبی در مقایسه با وضعیت خنثی و بدون استفاده از آن، به‌طور معناداری (0/043=P) افزایش یافت، ولی دیگر متغیرهای تعادل در این وضعیت افزایش معناداری نداشتند. هیچگونه تفاوت معناداری بین متغیر‌های مختلف تعادل در وضعیت خنثی سر و گردن، با استفاده از گردنبند طبی و بدون استفاده از آن دیده نشد. همچنین کاهش معناداری در ثبات وضعی در حالت 20 درجه مایل شدن سر و گردن به عقب وجود نداشت. نتیجه‌گیری: استفاده از گردنبند طبی در حالت به‌عقب‌خم‌کردن (اکستانسیون) سر و گردن باعث کاهش ثبات وضعی می‌گردد، درحالی‌که استفاده از آن در وضعیت خنثی (نوترال) تأثیر حادی بر ثبات وضعی ندارد.

Title: بررسی تکرارپذیری متغیرهای مرکز فشار در نوسان ایستایی با سطوح مختلف خستگی و بینایی در مردان ورزشکار و غیرورزشکار

http://rehabilitationj.uswr.ac.ir/article-1-662-fa.html

هدف: تحقیق حاضر به بررسی تفاوتهای مرتبط با مهارت، خستگی و بینایی بین ورزشکاران و غیرورزشکاران در تکرارپذیری اندازه‌های مرکز فشار حاصل از صفحه نیرو می‌پردازد. روش بررسی: در این مطالعه اعتبارسنجی، نوسان ایستایی ۱۵ ورزشکار مرد و ۱۶ غیرورزشکار مرد که به‌صورت ساده و در دسترس انتخاب شده بودند، توسط صفحه نیرو ارزیابی شده و پس از گذشت 48 تا 72 ساعت، ارزیابی مجدد صورت گرفت. متغیرهای مرکز فشار در خلال ایستادن ثابت روی دو پا، قبل و بعد از ایجاد خستگی عمومی توسط نوارگردان، با چشمان باز و بسته ثبت شد. اطلاعات حاصل از 30 ثانیه آزمون روی صفحه نیرو، برای متغیرهای انحراف معیار سرعت، انحراف معیار جابجایی در هر دو نمای قدامی– خلفی و جانبی و سرعت متوسط محاسبه شد. جهت تجزیه و تحلیل اطلاعات از آزمون تی مستقل و محاسبه ضریب همبستگی درون‌گروهی و خطای استاندارد اندازه‌گیری و ضریب تغییر استفاده شد. یافته‌ها: ضریب همبستگی درون‌گروهی برای اندازه‌های مرکز فشار در گروه ورزشکاران در مقایسه با غیرورزشکاران مقادیر بیشتری را نشان داد. پس از خستگی ضریب همبستگی درون‌گروهی نسبت به قبل از آن افزایش یافت. همچنین ضریب همبستگی درون‌گروهی در حالت چشمان بسته در مقایسه با چشمان باز مقادیر بیشتری را نشان داد. ضریب تغییر کوچکتر از 15% برای اکثر اندازه‌های مرکز فشار مشاهده شد. سرعت متوسط (P=0. 001) و انحراف معیار سرعت در نمای قدامی– خلفی (P=0. 001)، تکرارپذیرترین متغیرها بر اساس سطح مهارت، خستگی و بینایی بودند. نتیجه‌گیری: نتایج پژوهش حاضر می‌تواند به محققان برای انتخاب متغیرهای مرکز فشار در تحقیقات بعدی کنترل ایستایی (پاسچر) در ورزشکاران کمک کند. بر این اساس می‌توان از متغیرهای سرعت متوسط و انحراف معیار سرعت در نمای قدامی– خلفی، در مطالعات بعدی، در شرایط مشابه و در گروه ورزشکاران استفاده نمود.
